# Hyperoncotic colloids and acute kidney injury: a meta-analysis of randomized trials

**DOI:** 10.1186/cc9308

**Published:** 2010-10-28

**Authors:** Christian J Wiedermann, Stefan Dunzendorfer, Luigi U Gaioni, Francesco Zaraca, Michael Joannidis

**Affiliations:** 1 Department of Internal Medicine, Central Hospital of Bolzano, Lorenz Böhler Street 5, 39100 Bolzano, Italy; 2 Department of Internal Medicine I, Medical University of Innsbruck, Anichstrasse 35, 6020 Innsbruck, Austria; 3 Department of Vascular and Thoracic Surgery, Central Hospital of Bolzano, Lorenz Böhler Street 5, 39100 Bolzano, Italy

## Abstract

**Introduction:**

It has been hypothesized that hyperoncotic colloids might contribute to acute kidney injury (AKI). However, the validity of this hypothesis remains unclear.

**Methods:**

A meta-analysis was conducted of randomized controlled trials evaluating AKI after infusion of hyperoncotic albumin and hydroxyethyl starch (HES) solutions. Mortality was a secondary endpoint. Eligible trials were sought by multiple methods, and the pooled odds ratios (OR) for AKI and death and 95% confidence intervals (CI) were computed under a random effects model.

**Results:**

Eleven randomized trials with a total of 1220 patients were included: 7 evaluating hyperoncotic albumin and 4 hyperoncotic HES. Clinical indications were ascites, surgery, sepsis and spontaneous bacterial peritonitis. Hyperoncotic albumin decreased the odds of AKI by 76% (OR, 0.24; CI, 0.12-0.48; *P *< 0.0001), while hyperoncotic HES increased those odds by 92% (OR, 1.92; CI, 1.31-2.81; *P *= 0.0008). Parallel effects on mortality were observed, with hyperoncotic albumin reducing the odds of death by 48% (OR, 0.52; CI, 0.28-0.95; *P *= 0.035) and hyperoncotic HES raising those odds by 41% (OR, 1.41; CI, 1.01-1.96; *P *= 0.043).

**Conclusions:**

This meta-analysis does not support the hypothesis that hyperoncotic colloid solutions *per se *injure the kidney. Renal effects appear instead to be colloid-specific, with albumin displaying renoprotection and HES showing nephrotoxicity.

## Introduction

The potential for beneficial or deleterious renal effects is a key consideration in the selection and use of colloid solutions for clinical fluid management. In a systematic review, albumin was found to protect the kidney, whereas the carbohydrate-based artificial colloids hydroxyethyl starch (HES) and dextran were frequently associated with acute kidney injury (AKI) [1]. Confirmation with respect to the negative effects of HES has been provided by two recent meta-analyses of randomized trials showing increased incidence of acute renal failure (ARF) [2] and need for renal replacement therapy (RRT) [3] in patients receiving HES and by a systematic review [4].

Renal effects might be influenced not only by the specific properties of the particular colloid molecule but also by higher colloid osmotic pressure (COP) [5], assuming that increased COP decreases effective glomerular filtration pressure and thus glomerular filtration rate antagonizing hydrostatic pressure [6]. Colloid solutions can be classified as hypo-oncotic, iso-oncotic, or hyperoncotic according to whether their COP is less than, similar to, or greater than that of plasma, respectively. COP is strongly dependent upon the concentration of colloid in the solution [7]. Thus, 4% to 5% albumin is hypo-oncotic, whereas 20% to 25% albumin is hyperoncotic [8-11]. Similarly, 6% HES is iso-oncotic, whereas 10% HES is hyperoncotic [8,9,11,12]. Molecular weight and substitution show little if any effect on the COP of HES solutions [9,11].

With their capacity to draw interstitial fluid into the intravascular compartment, hyperoncotic solutions provide an attractive option for volume expansion because they are rapidly effective in a small infused volume and can serve to minimize edema [13]. However, in an analysis of data from the CRYstalloids or COlloids (CRYCO) study observational study of 1,013 intensive care unit patients, exposure to hyperoncotic solutions of either albumin or HES was associated with increased occurrence of AKI as compared with crystalloids or hypo-oncotic colloids [5]. That analysis suggests that hyperoncotic colloids *per se *might be harmful to the kidney. On the other hand, no adverse renal effects were evident in a multicenter study of 600 patients receiving hyperoncotic 25% albumin [14] or in a recent meta-analysis of 25 randomized trials (with a total of 1,485 patients) evaluating hyperoncotic albumin [13]. Moreover, in randomized trials, iso-oncotic 6% HES increased the incidence of ARF in patients with severe sepsis or septic shock [15] and the need for RRT after kidney transplantation [16]. In light of the conflicting data, investigators are uncertain about the renal effects of hyperoncotic colloids. The present meta-analysis of randomized trials was designed to test the hypothesis that hyperoncotic colloids *per se *increase the incidence of AKI.

## Materials and methods

### Endpoints

This meta-analysis addressed the question: does infusion of hyperoncotic 20% to 25% albumin or 10% HES to prevent or correct hypovolemia increase the risk of AKI as compared with crystalloid, hypo-oncotic 4% to 5% albumin, or no fluid? Mortality was a secondary endpoint.

### Study selection

Parallel-group randomized controlled trials (RCTs) were eligible for inclusion if they evaluated the occurrence of AKI in patients receiving hyperoncotic solutions of 20% to 25% albumin or 10% HES for prevention or correction of hypovolemia. The control regimen could consist of crystalloid, hypo-oncotic 4% to 5% albumin, or no fluid but not a non-fluid intervention such as an active drug or a procedure. Hypo-oncotic colloids were also included as control fluids in the CRYCO study [5]. Trials employing an iso-oncotic 6% HES control arm or comparing two hyperoncotic colloids were excluded. The difference in colloid osmotic pressure (COP) between hyperoncotic colloids and iso-oncotic 6% HES of 12 to 18 mm Hg is smaller than that between hyperoncotic colloids and hypo-oncotic 4% to 5% albumin [8,9]; as a consequence of the smaller difference, AKI and mortality comparisons versus iso-oncotic 6% HES as a control fluid would likely be relatively insensitive. Colloids were classified as hyperoncotic on the basis of final concentration at the time of use; hence, cardiac surgery trials of extracorporeal circuit priming in which hyperoncotic colloid was extensively diluted prior to use were not eligible. No restrictions were placed on trial time period or reporting language. Both published and unpublished trials were sought.

### Search strategy

Computer searches were performed between April and July 2010 in Medline, Embase, records of published and unpublished trials in the Cochrane Library, the ClinicalTrials.gov website, and the abstract databases from major meetings in surgery, anesthesiology, intensive care, and hepatology. The search terms of inclusion were the following: kidney, renal, renin, mortality, injury, failure, complication, adverse, illness, outcome, cirrhosis, albumin, hetastarch, pentastarch, pentaspan, hyperoncotic, 10%, 20%, 25%, clinical trials, prospective studies, fluid therapy, random allocation, and humans. The search terms of exclusion were cohort study, observational, survey, pharmacokinetic, retrospective studies, RCTs as topic, practice guidelines as topic, animal, rats, pigs, swine, review, news, letter, comment, editorial, meta-analysis, hypervolemia, molecular adsorbent recirculating system [MARS], MARS, albumin-bound, chemotherapy, paclitaxel, methotrexate, nanoparticle, and microsphere. Roots and variants of the search terms were also used. Eligible trials were also sought by examining the reference lists of primary study publications and review articles and contacting resuscitation fluid suppliers. All investigators participated in determining the eligibility of candidate trials. Differences in interpretation were resolved through discussion.

### Data extraction

The authors, time periods, patients, and methods of each trial report were scrutinized to avoid duplication and ensure the most complete possible data set. From the reports of the included studies, the following data were extracted: year reported, number of patients, clinical indication, blinding, allocation concealment, patient age and gender, fluid regimen, criteria for diagnosing AKI, and incidence of AKI and death on the basis of intent to treat.

### Statistical analysis

The pooled odds ratios (ORs) for AKI and mortality and their 95% confidence intervals (CIs) were computed under a random effects model. Heterogeneity was evaluated by Cochran Q test and calculation of the I^2 ^statistic and publication bias by linear regression of standardized effect in relation to precision. Study quality was judged on the basis of blinding and allocation concealment. Analysis was performed with Comprehensive Meta Analysis version 2.2.048 (Biostat, Inc., Englewood, NJ, USA) statistical software.

## Results

### Included trials

**Figure 1:**
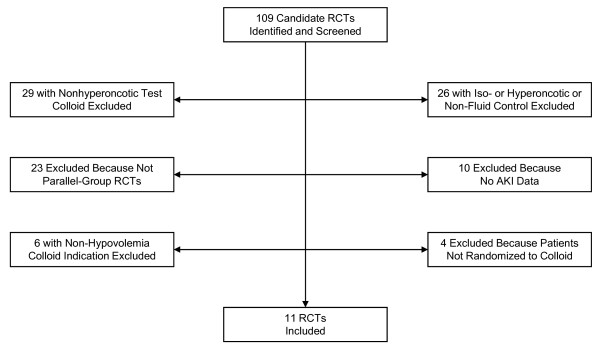
**Randomized controlled trial (RCT) selection process**. AKI, acute kidney injury.

The number of candidate RCTs identified and screened was 109 (Figure 1). Upon detailed evaluation of the candidates, 98 were excluded. The most common reasons for exclusion were a non-hyperoncotic test colloid, an iso- or hyperoncotic colloid, a drug or procedure control, and an investigational design other than a parallel-group RCT. Eleven RCTs with a total of 1,220 patients fulfilled all eligibility criteria and were included in the meta-analysis [17-27]. All trials had been published. Three of the 11 included trials were reported in the 1980 s and four each in the 1990 s and 2000 s (Table 1). Two trials were blinded and two were not, whereas the use of blinding was unspecified for the remaining seven trials. Allocation concealment was adequate in five trials and unspecified in six. The indication for volume expansion with colloid was treatment of ascites as an adjunct to paracentesis in three trials and to diuretics in one, surgery in three, and sepsis and spontaneous bacterial peritonitis in two each. One trial encompassed separate protocols for acute treatment of ascites in the hospital and subsequent long-term outpatient maintenance therapy [22]. Only the results from the acute treatment protocol were used in the meta-analysis. The median number of patients per trial was 72, and the interquartile range (IQR) was 32 to 116. More than 100 patients were evaluated in four trials, but only one trial involved over 200 patients. With 537 patients, that trial [25] was by far the largest included in the meta-analysis. Mean ages of patients in the trials were comparatively homogeneous. Whereas age averaged 49.1 years in one trial, the mean values in the other 10 trials fell within the relatively narrow range of 55.7 to 64.7 years. The median percentage of male patients in the included trials was 60% (IQR 60% to 65%). Seven trials evaluated hyperoncotic 20% to 25% albumin, and four trials evaluated hyperoncotic 10% HES. Control treatments were no colloid in seven trials, crystalloid in three, and hypo-oncotic colloid in one.

**Table 1:** Included randomized trials

Trial	Indication	Mean age, years	Treatment	AKI criteria
Zetterström and Hedstrand [17], 1981	Elective major abdominal surgery	58.5	Postoperative crystalloid with versus without 20% albumin	At least 70% SCr and urea increase
Ginès et al. [18], 1988	Treatment of ascites in hospitalized cirrhotic patients	57.0	Paracentesis with versus without 20% albumin	At least 50% SCr or BUN increase to greater than 1.5 or 30 mg/dL, respectively
London et al. [19], 1989	CPB surgery	63.5	10% pentastarch versus 5% albumin for first 24 hours postoperatively	Emergency dialysis necessitated by acute renal failure
García-Compeán et al. [20], 1993	Hospital treatment of tense ascites causing respiratory dysfunction in cirrhotic patients	55.7	Total therapeutic paracentesis with versus without 25% albumin	Greater than 50% SCr or BUN increase to greater than 1.5 mg/dL or greater than 30 mg/dL, respectively
Dehne et al. [21], 1997	Hypovolemia in surgical ICU patients	49.1	Normocaloric parenteral nutrition with versus without 12 mL/kg per day HES 200/0.5	Acute renal failure
Gentilini et al. [22], 1999	Hospital treatment of ascites in cirrhotic patients unresponsive to bed rest and low-sodium diet	62.1	Diuretics with versus without 50 mL 25% albumin daily	Acute renal failure
Sort et al. [23], 1999	Spontaneous bacterial peritonitis	61.0	Intravenous cefotaxime with versus without 1.5 g/kg 20% albumin on day 1 plus 1.0 g/kg on day 3	Greater than 50% SCr or BUN increase and, in patients without pre-existing renal failure, greater than 1.5 mg/dL SCr or greater than 30 mg/dL BUN
Sola-Vera et al. [24], 2003	Prevention of paracentesis-induced circulatory dysfunction in cirrhotic patients with ascites	61.4	20% albumin versus saline starting 3 hours after paracentesis	Greater than 100% SCr increase to greater than 2 mg/dL
Brunkhorst et al. [25], 2008	Severe sepsis or septic shock	64.7	10% pentastarch versus Ringer's lactate	Need for renal replacement therapy^a^
McIntyre et al. [26], 2008	Early septic shock	63.3	10% pentastarch versus normal saline	Requirement for dialysis during hospitalization
Chen et al. [27], 2009	Spontaneous bacterial peritonitis	56.5	Cephalosporins with versus without 50 mL 20% albumin on days 1 to 3	Greater than 50% SCr increase and, in patients without pre-existing renal failure, greater than 1.5 mg/dL SCr

^a^Need based on the presence of acute renal failure or another indication such as volume overload or hyperkalemia. Acute renal failure, defined as a doubling of the baseline serum creatinine (SCr) level or the need for renal replacement therapy, was evaluated as an additional separate endpoint in this trial. For the meta-analysis, only the data for renal replacement therapy were used. AKI, acute kidney injury; BUN, blood urea nitrogen; CPB, cardiopulmonary bypass; HES, hydroxyethyl starch; ICU, intensive care unit.

### Acute kidney injury

**Figure 2:**
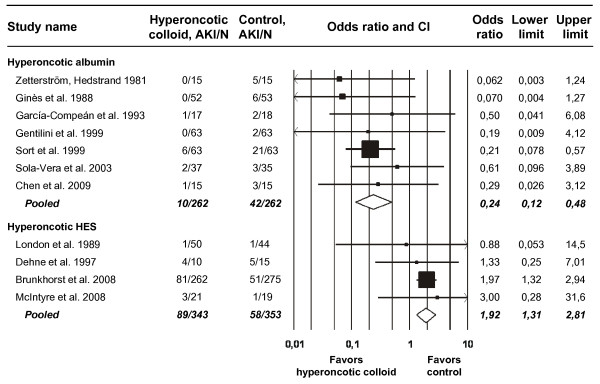
**Meta-analysis of acute kidney injury (AKI) after hyperoncotic colloid administration**. Data points are scaled in proportion to meta-analytic weight. Error bars indicate confidence interval (CI). HES, hydroxyethyl starch; N, total number of patients.

The AKI diagnosis criteria adopted in four trials included an increase of 50% or more in serum creatinine or blood urea nitrogen (Table 1). The need for RRT was the criterion in three trials, whereas other or unspecified criteria were applied in two trials each. Across all 11 studies, 199 of 1,220 patients (16%) developed AKI (Figure 2). As shown in Figure 2, hyperoncotic albumin decreased the odds of AKI by 76% (OR 0.24, CI 0.12 to 0.48; *P *< 0.0001). Among the seven trials evaluating hyperoncotic albumin, there was no evidence of either heterogeneity (*P *= 0.81; I^2 ^= 0%) or publication (*P *= 0.74) bias with respect to the AKI endpoint. Hyperoncotic HES showed the opposite effect (Figure 2), increasing the odds of AKI by 92% (OR 1.92, CI 1.31 to 2.81; *P *= 0.0008). Neither heterogeneity (*P *= 0.89; I^2 ^= 0%) nor publication (*P *= 0.55) bias was detectable in the AKI data from the four trials of hyperoncotic HES.

### Mortality

**Figure 3:**
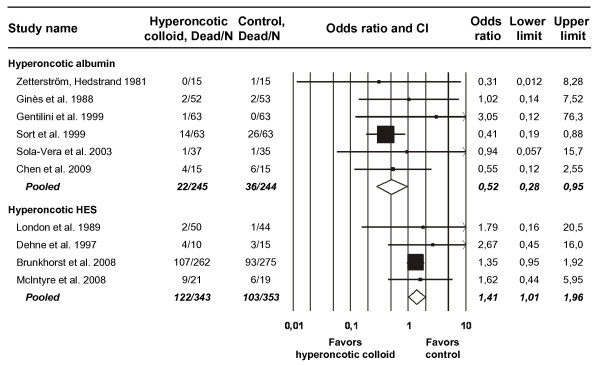
**Meta-analysis of mortality after hyperoncotic colloid administration**. Graphic conventions are the same as in Figure 2. CI, confidence interval; HES, hydroxyethyl starch; N, total number of patients.

Among all 11 included trials, 283 of 1,220 patients (23%) died. At least one death occurred in 10 of the 11 included trials, allowing those 10 trials (with a total of 1,185 patients) to be included in a meta-analysis evaluating the OR for mortality (Figure 3). The only trial with no deaths assessed hyperoncotic albumin in the treatment of ascites [20]. Hyperoncotic albumin reduced the odds of mortality by 48% (OR 0.52, CI 0.28 to 0.95; *P *= 0.035; Figure 3). No significant heterogeneity (*P *= 0.81; I^2 ^= 0%) or publication (*P *= 0.12) bias was present regarding mortality in the six trials of hyperoncotic albumin with at least one death. Conversely, hyperoncotic HES raised the odds of mortality by 41% (OR 1.41, CI 1.01 to 1.96; *P *= 0.043). The mortality data in the hyperoncotic HES trials did not display either heterogeneity (*P *= 0.89; I^2 ^= 0%) or publication (*P *= 0.14) bias.

## Discussion

This meta-analysis of RCTs did not support the hypothesis that hyperoncotic colloids *per se *are injurious to the kidney. It appeared that, within physiologic ranges, the specific properties of the colloid molecule rather than concentration are the major determinants of renal effects. According to our results, administration of hyperoncotic albumin was associated with reduced risk of AKI as well as with improved survival. By contrast, HES displayed nephrotoxicity and worsened survival.

Certain limitations of this meta-analysis should be noted. Criteria for diagnosing AKI were not standardized. Other than one surgery trial, all six other included trials of hyperoncotic albumin involved cirrhotic patients, whereas those evaluating hyperoncotic HES all concerned surgery or sepsis. Thus, the clinical settings for evaluation of the two hyperoncotic colloids were largely non-overlapping.

Six of the seven included trials evaluating hyperoncotic albumin involved cirrhotic patients. In all six of those trials, the observed reduction in the AKI incidence was less than in the seventh trial, which evaluated major abdominal surgery patients [17]. Extravascular fluid accumulation is a common complication of cirrhosis, which might be precipitated or exacerbated by hypo-oncotic fluid, and in five of the six included trials of cirrhotic patients, the selected control regimen consisted of no volume expander. The possibility could be entertained that volume expansion with a control fluid might have produced the same results as did hyperoncotic albumin in those trials. In one included trial [24], however, cirrhotic patients with ascites did receive saline as the control fluid. That group developed paracentesis-induced circulatory dysfunction with a frequency that was significantly higher (33.3%) than that of the patients allocated to hyperoncotic albumin (11.4%). Additionally, a randomized trial not included in this meta-analysis compared hyperoncotic 20% albumin with iso-oncotic 6% HES 200/0.5 in cirrhotic patients with spontaneous bacterial peritonitis [28]. Although the trial was not powered to assess AKI, the incidence of AKI was nevertheless lower in the hyperoncotic albumin group (OR 0.29, CI 0.03 to 3.12), and significant improvement in circulatory function was demonstrated in that group but not among the patients assigned to iso-oncotic HES.

The present meta-analysis is the first to investigate AKI specifically after infusion of hyperoncotic 10% HES. Prior systematic reviews and meta-analyses have not differentiated between iso-oncotic and hyperoncotic HES solutions [1-3,29]. Even in the CRYCO analysis, the designated hyperoncotic HES group did not actually receive hyperoncotic solutions exclusively [5]. Iso-oncotic 6% HES 130/0.4, for example, was among the solutions assigned to the CRYCO hyperoncotic HES group.

In three of the four included trials evaluating hyperoncotic HES, AKI was more frequent in the hyperoncotic HES group than the control group, and in all four of those trials, mortality was higher in hyperoncotic HES recipients. Nevertheless, the preponderance of the statistical power was derived from a single large trial [25], and it should be recognized that the conclusions of the meta-analysis regarding hyperoncotic HES rest primarily on that trial. If that trial were to be excluded, the point estimates of the pooled ORs for AKI (1.53) and mortality (1.91) would be comparable to those without the exclusion (1.92 and 1.41, respectively); however, the effects would no longer be statistically significant.

While this meta-analysis has shown increased risk of AKI because of hyperoncotic HES, randomized trials have demonstrated similar deleterious renal effects in patients receiving iso-oncotic HES [15,16]. Furthermore, in the CRYCO analysis, the incidence of AKI among recipients of iso-oncotic 6% HES 130/0.4 was similar to that of recipients of other evaluated HES solutions.

The present finding of renal protection attributable to hyperoncotic albumin in randomized trials is in contrast to the report of increased AKI in the hyperoncotic albumin group from the CRYCO analysis. Several factors may limit the reliability and generalizability of the CRYCO results with respect to hyperoncotic albumin, namely use of hyperoncotic albumin in a small minority of the most severely ill patients, concomitant infusion of other colloids, absence of a demonstrated dose-response relationship, and exclusion of cirrhotic patients.

No evidence of adverse renal effects was uncovered in a multicenter study of 600 patients receiving 25% albumin [14]. In that observational study, which was specifically designed to evaluate safety, normothermic hypoproteinemic patients at 14 US hospitals received multiple infusions of 80 to 100 mL of 25% albumin over a maximum period of 570 days. Forty-four patients underwent more than 10 infusions each, and the cumulative dose administered to five patients exceeded 800 g. The patients were closely monitored for adverse events, including pyrogenic reactions, anaphylactoid and cardiovascular symptoms, and pulmonary edema. Post-mortem examination of 16 patients who had received 25% albumin doses up to 813 g each showed no evidence of abnormal albumin storage or other renal abnormalities that could not be explained by the disease of which the patients died.

On the basis of the CRYCO findings, recently issued clinical guidelines recommended the avoidance of hyperoncotic dextran, HES, and albumin solutions because of the risk for renal dysfunction [30]. In light of this meta-analysis and the limitations of the CRYCO study, such a blanket recommendation would appear to be unwarranted. A more differentiated approach to the use of these solutions is needed, as recommended in a recent publication [4].

Albumin-mediated renoprotection may be explained by several mechanisms, including maintaining renal perfusion [6,31,32], promoting proximal tubular integrity and function [22,33-38], binding of endogenous toxins and nephrotoxic drugs [39-42], and preventing oxidative damage and binding and delivering protective lysophosphatidic acid [34,36,43,44]. The important renoprotective role of serum albumin was underscored by a recent meta-analysis showing hypoalbuminemia to be a potent independent risk factor both for AKI and for death following the development of AKI [45].

Many medications have been associated with toxic effects on the kidney [46]. Because the kidney receives a quarter of resting cardiac output, glomerular, tubular, and renal interstitial cells can be exposed to substantial concentrations of medications and their metabolites, which can induce changes in kidney structure and function. On the other hand, several mechanisms of HES-induced AKI are reported. HES is degraded and its degradation products are reabsorbed mainly in the proximal tubule. Intracellular accumulation resulting in vacuolization of the proximal tubule may result in functional impairment. Post-mortem examination of 12 patients who received repeated HES 200/0.5 infusions and died after protracted RRT because of ARF showed that the kidney contained the highest tissue concentration of HES compared with any of the other six major organs evaluated [47]. Degradation products of HES are cleared primarily via the kidney. Some of these breakdown products are excreted in the urine, but others can be taken up by the cells of the proximal tubule through pinocytosis. The pinocytotic vacuoles subsequently fuse with each other and lysosomes to form larger vacuoles, which can displace other cellular components. HES can accumulate through storage in these oversized lysosomes. There have been no studies published thus far on the type of HES metabolites taken up in the vacuoles and the ability of lysosomal enzymes to degrade them. Vacuolization and swelling of the renal proximal tubular cells are often harbingers of AKI [48].

The histopathologic pattern of acute tubular injury typical of HES accumulation in the kidney is osmotic nephrosis. This phenomenon was first described in 1940 when patients with increased intracranial pressure were treated with intravenous sucrose solutions [48]. Dextran, another colloid composed of glucose units, has also been associated with osmotic nephrosis [1], as have maltose and mannitol [48]. Numerous case reports have documented osmotic nephrosis in patients developing AKI after HES exposure [49-52]. In a randomized trial, HES increased the need for RRT after kidney transplantation, and all biopsied patients receiving HES showed osmotic nephrosis, but none in the control group did [16]. Hydroxyethylation of constituent glucose molecules in the synthesis of HES is specifically intended to retard degradation and prolong action. Osmotic nephrosis resulting from HES exposure can be extremely long-lasting, if not permanent. During long-term follow-up after orthotopic liver transplantation, osmotic nephrosis attributable to HES persisted up to 10 years in 61% of patients [53].

Decreasing levels of renoprotective albumin might be an additional mechanism underlying the nephrotoxicity of HES, which has been shown to cause iatrogenic hypoalbuminemia [54-59]. Inflammatory processes may also contribute to HES-mediated AKI [60]. Further research is needed on pathophysiologic mechanisms as well as on the metabolic fate of HES in the kidney.

Only relatively recently has attention been focused on the possibility that hyperoncotic fluids *per se *might be injurious to the kidney. Accordingly, the hypotheses tested in the trials of this meta-analysis were not formulated specifically in terms of evaluating a hyperoncotic colloid. Nevertheless, in those trials, patients were randomly allocated to a hyperoncotic colloid or control regimen, and the trials therefore do provide relevant evidence of the renal effects of hyperoncotic colloids. It has recently become increasingly common to discuss 'hyperoncotic acute renal failure'. This meta-analysis raises doubt that such a concept can satisfactorily account for AKI in patients receiving colloids.

## Conclusions

Currently available randomized trial evidence suggests that hyperoncotic albumin solutions may reduce the incidence of AKI and death. The opposite effects appear to be exerted by hyperoncotic HES.

## Key messages

• It has been hypothesized that hyperoncotic colloid solutions may damage the kidney. A meta-analysis of randomized controlled trials was performed to test this hypothesis.

• Hyperoncotic albumin decreased the odds of acute kidney injury by 76% and of death by 48%.

• Hyperoncotic hydroxyethyl starch increased the odds of acute kidney injury by 92% and of death by 41%.

• Hyperoncotic colloids *per se *do not appear to be harmful to the kidney.

• Renal effects may be specific to the particular colloid molecule.

## Abbreviations

AKI: acute kidney injury; ARF: acute renal failure; CI: confidence interval; COP: colloid osmotic pressure; CRYCO: CRYstalloids or Colloids; HES: hydroxyethyl starch; IQR: interquartile range; OR: odds ratio; RCT: randomized controlled trial; RRT: renal replacement therapy.

## Competing interests

CJW has received speaker fees and travel reimbursements from manufacturers of plasma-derived therapies (CSL Behring, King of Prussia, PA, USA, and Kedrion, Prato, Italy). The other authors declare that they have no competing interests.

## Authors' contributions

CJW conceived the meta-analysis and participated in its design and coordination, extracted data, performed statistical analysis, contributed to the interpretation of results, and helped to draft the manuscript. MJ participated in the design and coordination of the meta-analysis, contributed to the interpretation of results, and helped to draft the manuscript. All authors participated in searching for trials and determining their eligibility for inclusion in the meta-analysis. All authors read and approved the final manuscript.

## Authors' information

CJW and MJ were co-authors of a recent meta-analysis on hypoalbuminemia and acute kidney injury. CJW authored a systematic review on the use of hydroxyethyl starch for fluid management in sepsis. MJ first-authored the publication of an expert opinion statement of the European Society of Intensive Care Medicine Working Group for Nephrology on renal protection in the intensive care unit.
